# Attenuation of hexaconazole induced oxidative stress by folic acid, malic acid and ferrocenecarboxaldehyde in an invertebrate model *Bombyxmori*

**DOI:** 10.1016/j.heliyon.2022.e12577

**Published:** 2022-12-26

**Authors:** Hashim Ashraf, Ayesha Qamar, Nikhil Maheshwari

**Affiliations:** a Section of Entomology, Department of Zoology, Aligarh Muslim University, Aligarh 202002, India; b Department of Biochemistry, Aligarh Muslim University, Aligarh 202002, India

**Keywords:** Hexaconazole, Folic acid, Malic acid, Ferrocenecarboxaldehyde, Oxidative stress, *Bombyx mori*

## Abstract

Fungicides are a class of pesticides used to ward off fungal diseases from agricultural crops to achieve maximum productivity. These chemicals are quite efficient in controlling diseases; however, the excessive use of these affects non-target organisms as well. In this study, *Bombyx mori* was utilized to investigate the effect of the pesticide hexaconazole (HEX) on the antioxidant system of this organism and also to find ways to mitigate it. On oral exposure to this chemical, a significant reduction in antioxidants, CAT, GPX, GSH, and SOD in the gut, fat body, and silk gland was observed. The HEX treatment also resulted in lipid peroxidation (LPO) in all the three tissues. To mitigate this toxicity and protect the silkworm from oxidative stress, we tested three compounds, namely folic acid, ferrocenecarboxaldehyde, and malic acid having known antioxidant potential. Folic acid provided significant protection against HEX-induced toxicity. Ferrocenecarboxaldehyde and malic acid proved to be ill-efficient in controlling oxidative stress, with ferrocenecarboxaldehyde being the least effective of the three. Folic acid was also efficient in controlling LPO up to a considerable level. Ferrocenecarboxaldehyde and malic acid also prevented LPO less efficiently than folic acid. Overall folic acid was the only compound that mitigated HEX-induced oxidative stress in silkworm with statistical significance in all the tissues *viz*. gut, fat body, and silk gland.

## 1 Introduction

Pesticides are used to keep pests out of crops and prevent diseases and infections. In the modern world, the use of pesticides has become essential to achieving economically viable crop production. Without pesticides, present agriculture will not be able to meet the needs of the globe (Nicolopoulou-Stamati et al., 2016; Popp et al., 2013; Tudi et al., 2021). However, pesticides are hazardous compounds that, despite being used on a specific target, also have adverse effects on organisms that are not the intended target (Gao et al., 2020; Neuwirthová et al., 2019). The most susceptible organisms to this toxicity are non-target organisms, particularly natural insect predators. In controlling insect populations, natural enemies are crucial. Therefore, any detrimental impact on these organisms would allow pest populations to grow, necessitating more pesticide spraying to keep them under control (Gill and Garg, 2014; Sánchez-Bayo, 2021). Exposure to pesticides has been related to cancer (Navarrete-Meneses and Pérez-Vera, 2019), Parkinson's disease (Cagac, 2020; Liu et al., 2020), tuberculosis, chronic respiratory illnesses, liver disorders (Wahlang et al., 2020), DNA damage (Mehdi & Qamar, 2013) and alterations in thyroid TSH (Thyroid stimulating hormone) levels in people (Bernieri et al., 2019; Bhat et al., 2010).

The selected pesticide, Hexaconazole (HEX), is a member of the triazole family of fungicides. It works by inhibiting the 14 α -demethylase enzyme, which is mediated by cytochrome P450, preventing the synthesis of fungal sterols (Worthington, 1991). HEX has been reported to cause toxicity in numerous non target organisms in various studies. HEX exposure caused a decrease in antioxidant enzyme concentrations, bio-accumulation, endocrine disrupting effect (zebrafish) (Yu et al., 2013), up-regulated apoptotic pathway genes, altered lipid, amino acid, and energy metabolism (zebrafish) (Jia et al., 2019). HEX causes genotoxicity by producing structural and numerical abnormalities in mouse cells and mammalian cells (Yilmaz et al., 2008), and affects lipid-related pathways (mouse) (Sun et al., 2021). In humans, HEX poisoning has been documented, causing neurotoxic effects (trembling, jittering, and shaking) and vomiting/nausea (Acharya and Panda, 2021; David et al., 2008).

To protect body from oxidative stress induced by xenobiotics like pesticide and other toxic compounds, organisms have evolved a mechanism where antioxidant enzymes function in synergy and protect the body from oxidative stress, however, to a certain extent only. Antioxidant enzymes are essential for maintaining a healthy equilibrium between the production of reactive oxygen species (ROS) and their degradation. An increase in the number of ROS produced inside of a cell can cause oxidative stress and damage to DNA, lipids, and proteins (Chen et al., 2018; Jia et al., 2011; Lopez-Martinez et al., 2008). Any type of stress can upset the balance between ROS generation and degradation, resulting in the production of more ROS which then results in Lipid peroxidation (LPO) (Juan et al., 2021; Lalouette et al., 2011). Malondialdehyde (MDA) levels could be utilized to estimate LPO indirectly (Meng et al., 2009). The primary anti-oxidative enzymes that support maintaining ROS balance are superoxide dismutase (SOD), catalase (CAT), and glutathione S-transferases (GSTs) (Dubovskiy et al., 2008; Meng et al., 2009). While CAT converts hydrogen peroxide (H2O2) into water and oxygen, SOD, on the other hand, converts superoxide anion (O_2_^-^) into oxygen (O_2_) and hydrogen peroxide (H_2_O_2_). Glutathione (GSH) is a non-enzymatic antioxidant whose function is to protect cells by neutralizing ROS (Wu et al., 2004). Glutathione Peroxidase (GPx) a type of oxidoreductase that is dependent on selenium and uses H_2_O_2_ or organic hydroperoxide as the oxidant and uses the tripeptide GSH as the electron donor (Cardoso et al., 2017).

In addition to the antioxidant system that are already present in the organism, some substances that are both naturally occurring and synthetic can act as antioxidants and scavenge ROS in a variety of conditions. Therefore, to amplify the antioxidant response of an organism, antioxidant therapy works well in many instances. For this reason, we choose antioxidant compounds, folic acid, malic acid, and ferrocenecarboxaldehyde for mitigating the toxicities of HEX on silkworm *Bombyx mori* (*B. mori*)*.* Numerous studies point to the antioxidant potential of these compounds in scavenging ROS. According to a meta-analysis study, folic acid intake considerably improves antioxidant indicators by raising blood concentrations of GSH while lowering concentrations of MDA. This results in a considerable increase in total antioxidant capacity (TAC) and GSH levels (Asbaghi et al., 2021). Folic acid controlled and mitigated oxidative stress levels by targeting insulin/insulin growth factor 1 (IGF-1) signalling pathways in *Caenorhabditis elegans* (*C. elegans)* (Rathor et al., 2015), reduced LPO, and protected against the body weight changes induced by dexamethasone, oxidative stress, and cerebrovascular disease (rats) (Atteia et al., 2009; Cui et al., 2018; Li et al., 2016). Co-administration of folic acid with the pesticide cyhalothrin decreased its reproductive toxicity (mice) (Elnaggar et al., 2012).

To mitigate the toxicity induced by ROS stress, a chemical compound, ferrocene and its derivatives (synthetic ferrocene derivatives) have been proposed as a potential new class of antioxidants, showing free radical scavenging properties (DAS et al., 2022). Ferrocenecarboxaldehyde, a derivative of ferrocene has been shown to possess antioxidant activities. The antioxidant properties of ferrocenecarboxaldehyde were studied for free radical scavenging activity towards DPPH, Nitric oxide (NO), superoxide anion, and by UV-VIS and electron spin resonance spectroscopies and the result revealed a greater antioxidant potential of ferrocenecarboxaldehyde (Bugarinović et al., 2018; Jančić et al., 2022; Tabrizi et al., 2020). Another class of chemicals called organic acids has been reported to possess strong antioxidant properties. Organic acids are a good source of antioxidants. According to some studies, certain organic acids have a variety of pharmacological effects, including an anti-inflammatory response, antioxidant properties, and a reduction in cell death (Dharmappa et al., 2009; Varì et al., 2011; Wang et al., 2010). Malic acid is one of the organic acids commonly found in fruits and is the predominant organic acid in apple, pear, loquat, etc (Ma et al., 2018). Organic acid like Protocatechuic acid has been found to have strong *in-vitro* and *in-vivo* antioxidant activity (Li et al., 2011; Semaming et al., 2015)*.* It would therefore be advantageous to use them to minimize oxidative stress to mitigate oxidative damage. We also choose malic acid, an organic acid, as a candidate for attenuation of oxidative stress with the hypothesis that it will act as a strong antioxidant like other organic acids.

All the compounds, viz. folic acid, ferrocenecarboxaldehyde, and malic acid were tested for their antioxidant efficiency for mitigation of HEX-induced stress on our invertebrate model *B. mori*. The use of *B. mori* as a model organism in numerous toxicological studies is not new. It has been used in studies such as toxicity of drug candidates (Abdelli et al., 2018), nanoparticle toxicity (Mir et al., 2020), pesticide toxicity (Qadri et al., 2022), heat stress research (Mir & Qamar, 2018), drug screening (Miyazaki et al., 2012), medical research (Nwibo et al., 2015). It features characteristics that make it the perfect model organism, including large body size, a rapid generation rate, minimal ethical issues, minimal breeding costs, and a clear genetic background with a significant number of genes that are homologous to human genes (Meng et al., 2017).

The effect of HEX on non-target organisms is poorly studied. Therefore, this work was carried out to check the effect of HEX on the antioxidant system of an invertebrate model *B. mori*. The study was also carried out to check the antioxidant potential and efficiency of three antioxidant compounds, viz. folic acid, ferrocenecarboxaldehyde, and malic acid. Ferrocenecarboxaldehyde and malic acid as antioxidants, to the best of our knowledge, have been poorly researched so far. This study, therefore, attempted to find the effectiveness of these compounds in ROS mitigation.

## 2 Methods

### 2.1 Insect culture

Silkworm eggs/disease free laying's (DFLs) were procured from seed center and, after hatching, were cultured under standard conditions of temperature (25^o^C–28 °C), humidity (70%–80%), and photoperiod (18L:8D).

### 2.2 Chemicals

A commercial formulation of (±) Hexaconazole 5% SC was purchased from the pesticide market in Aligarh, UP, India. Folic acid (CAS number: 59-30-3)., L-malic acid (CAS number: 97-67-6), and ferrocenecarboxaldehyde (CAS number: 12093-10-6) were purchased from Sigma-Aldrich. Other compounds used in the biochemical analysis were of technical grade, too, and were purchsed from Sigma-Aldrich.

### 2.3 Treatment

**Figure 1:**
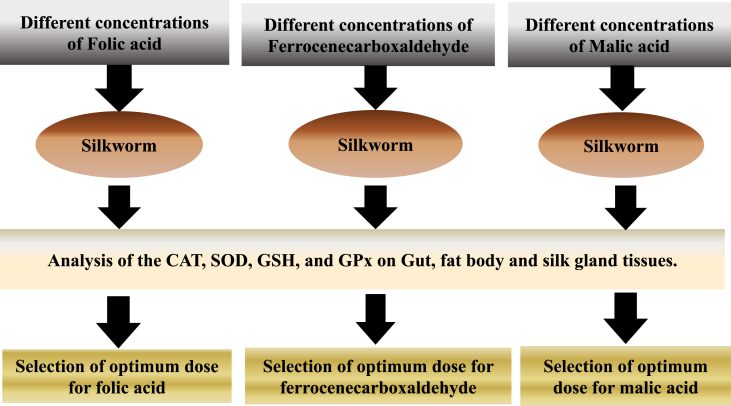
Schematic representation of the experimental design for selection of optimal doses of folic acid, ferrocenecarboxaldehyde and malic acid for attenuation of oxidative stress in *Bombyx mori*.

Different concentrations of HEX were prepared for studying the level of toxicity in silkworm. We chose a concentration of 100 mg/l for this study as this concentration was found to be sub-lethal, causing maximum antioxidative stress in our pilot study (S-1 & S-2). A 1% stock solution of folic acid (0.0362M), malic acid (0.1201M), and ferrocenecarboxaldehyde (0.0537M) were prepared from analytical grade folic acid, L-malic acid, and ferrocenecarboxaldehyde respectively. From the stock solution, working concentrations were prepared for the treatment. The toxicities of folic acid, malic acid, and ferrocenecarboxaldehyde on the antioxidant enzyme system in silkworm were evaluated before the selection of their final doses for this study Figure 1. For folic acid, a concentration of 0.018M was found to be best suited for mitigation purposes as this concentration did not hinder the functioning of antioxidant enzymes, and above this concentration, the concentration of antioxidants started declining Table 1. For malic acid, 0.0073M concentration was found to have the least impact on antioxidant enzyme working. Above this concentration, the level of antioxidants decreased in silkworm Table 2. For ferrocenecarboxaldehyde, 0.0054M was chosen as a candidate concentration for mitigation purposes as it was the optimum concentration with the least antioxidant level disturbances compared to the control set Table 3. The experiment was started by treating the silkworms with HEX *via.* oral route. Briefly, mulberry leaves were dipped in the HEX solution for 2 min, air-dried, and fed to the silkworms. After 30 min, the silkworms were fed again on mulberry leaves treated with folic acid (in T + F.A group), ferrocenecarboxaldehyde (in T + Fer group), and malic acid (in T + M.A group). Sampling in each group was done at 24 h, 48 h, and 72 h post-HEX treatment.

**Table 1:** Levels of CAT, SOD, GPx and GSH after treatment with antioxidants folic acid on Silkworm *B. mori*.

Biochemical test	Folic acid concentrations					
	control	0.0036M	0.0073M	0.0181M	0.0254M	0.0363M
**CAT activity**	6.125 ± 0.02	6.899 ± 0.06	6.258 ± 0.03	**6.873** ± 0.03	5.969 ± 0.05	3.968 ± 0.07
**SOD activity**	7.212 ± 0.01	6.965 ± 0.05	7.028 ± 0.03	**7.322** ± 0.01	7.008 ± 0.04	5.693 ± 0.03
**GPx activity**	3.227 ± 0.01	4.368 ± 0.03	3.146 ± 0.06	**3.281** ± 0.04	2.824 ± 0.01	1.865 ± 0.05
**GSH activity**	12.11 ± 0.03	12.47 ± 0.05	12.59 ± 0.02	**12.65** ± 0.03	7.66 ± 0.03	5.38 ± 0.04

**Table 2:** Levels of CAT, SOD, GPx and GSH after treatment with antioxidants malic acid on Silkworm *B. mori.*

Biochemical test	Malic acid concentrations					
	control	0.012M	0.024M	0.06M	0.084M	0.1201M
**CAT activity**	6.125 ± 0.01	5.128 ± 0.06	**5.882** ± 0.02	4.361 ± 0.03	3.256 ± 0.02	2.963 ± 0.05
**SOD activity**	7.212 ± 0.02	7.147 ± 0.07	**7.175** ± 0.04	7.008 ± 0.01	6.489 ± 0.05	5.442 ± 0.01
**GPx activity**	3.227 ± 0.01	3.108 ± 0.03	**3.147** ± 0.02	2.956 ± 0.03	2.433 ± 0.06	2.116 ± 0.06
**GSH activity**	12.11 ± 0.02	11.54 ± 0.07	**11.65** ± 0.03	10.89 ± 0.04	10.06 ± 0.04	9.627 ± 0.04

**Table 3:** Levels of CAT, SOD, GPx and GSH after treatment with antioxidants ferrocenecarboxaldehyde on Silkworm *B. mori.*

Biochemical test	Ferrocenecarboxaldehyde concentrations					
	control	0.0054M	0.017M	0.0268M	0.0376M	0.0537M
**CAT activity**	6.125 ± 0.03	**5.983** ± 0.08	5.886 ± 0.05	5.033 ± 0.04	4.986 ± 0.04	3.489 ± 0.04
**SOD activity**	7.212 ± 0.01	**6.892** ± 0.05	6.127 ± 0.05	6.109 ± 0.04	5.852 ± 0.02	4.731 ± 0.07
**GPx activity**	3.227 ± 0.01	**3.019** ± 0.06	3.256 ± 0.02	3.127 ± 0.09	3.068 ± 0.06	2.866 ± 0.05
**GSH activity**	12.11 ± 0.02	**11.231** ± 0.03	11.35 ± 0.05	10.49 ± 0.04	10.12 ± 0.03	9.817 ± 0.05

### 2.4 Biochemical analysis

#### 2.4.1 Preparation of tissue homogenates

*B. mori* had its fat body, gut, and silk gland removed at sampling periods of 24, 48, and 72 h These organs were subsequently washed in phosphate buffer and ground into a finer consistency. A 10% (w/v) tissue homogenate was made by with the help of a glass Teflon homogenizer and by delivering five pulses of 30 s each in a solution that included 2 mM Tris-HCl, 50 mM mannitol, and pH 7.0.

#### 2.4.2 Antioxidant enzymes and oxidative stress markers

To quantify proteins present in tissue homogenates, Lowry method was used (Lowry et al., 1951). By using the meta-phosphoric acid precipitation method, tissue homogenates were deproteinized. The resultant supernatants were used for GSH measurement using 5,5′-dithiobisnitrobenzoic acid (DTNB) following centrifugation at 15,000 rpm for 5 min at 4 °C (Beutler et al., 1963). LPO was measured by reaction of Malondialdehyde with the colouring agent thiobarbituric acid which gives a pink complex (Buege and Aust, 1978). SOD was measured from the suppression of auto-oxidation of pyrogallol, while the CAT assay is based on the enzymatic breakdown of H_2_O_2_ to H_2_O at 240 nm (Aebi, 1984) (Marklund and Marklund, 1974). GPx assay was measured by the decrease in the absorbance at 340 nm, which is a result of conversion of NADPH to NADP+ (Flohé and Günzler, 1984). The activity of every enzyme was observed in tissue homogenates.

## 3 Statistical analysis

Every experiment was run in triplicate (n = 20), and the significant differences between the control and treated groups with P < 0.05 were determined using one-way analysis of variance (ANOVA). Means between different groups were compared by post hoc Tukey's test. Software GraphPad Prism 5 was used for the statistical analysis.

## 4 **Results**

### 4.1 Biochemical analysis

#### 4.1.1 Gut

##### 4.1.1.1 CAT activity

**Figure 2:**
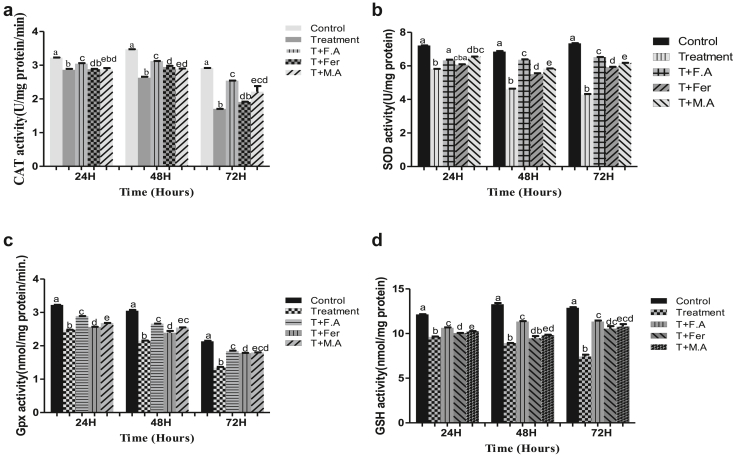
**A.** CAT activity in the gut of *B. mori* after hex treatment and antioxidant activity of folic acid, ferrocenecarboxaldehyde and malic acid. **B.** bar chart of SOD activity following hex treatment and effect of folic acid, ferrocenecarboxaldehyde and malic acid as antioxidant agents, **C.** GPx activity bar graph showing antioxidant activity of folic acid, ferrocenecarboxaldehyde and malic acid after hex treatment **D.** GSH activity bar graph after hex treatment and folic acid ferrocenecarboxaldehyde and malic acids efficiency as antioxidants. The bars with different superscript are significantly different, (P < 0.05).

With the treatment of HEX, compared to the control at 24 h, the treated group had a 10% decrease in CAT activity. At subsequent sampling times of 48 & 72 h, the CAT activity decreased to 24% and 41%, respectively, compared to the control group. Groups post-treated with folic acid had a 5% decline in CAT activity at 24 h, 10%, and 12% at 48 & 72 h, respectively. Groups post-treated with ferrocenecarboxaldehyde and malic acid recorded a CAT activity decline of 10%, 16.7%, and 34% at 24, 48, and 72 h respectively for ferrocenecarboxaldehyde and 10%, 16%, and 24% at 24, 48 & 72 h for malic acid (Figure 2A).

##### 4.1.1.2 SOD activity

SOD activity got reduced to 19% post-exposure to HEX at the sampling point 24 h and further reduced to 32% and 41% at sampling times of 48 & 72 h, respectively. Groups post-treated with the antioxidant compound folic acid, witnessed a SOD activity reduction of 12.2%, 3.1%, and 11% at sampling times of 24, 48 & 72 h, respectively. In a group post-treated with ferrocenecarboxaldehyde, SOD activity reduced to 16%, 19%, and 19% at periods of 24, 48, & 72 h, respectively. In the group post-treated with malic acid, there was a 9%, 14%, and 15% decline in SOD activity compared to the control group at sampling times of 24, 48, & 72 h, respectively (Figure 2B).

##### 4.1.1.3 GPx activity

A decrease in GPx activity was seen by HEX treatment, with the treatment group showing GPx decline of 23%, 29%, and 36% at sampling points of 24, 48, & 72 h, respectively. The decrease in the folic acid post-treated group was 10%, 12%, and 13% at sampling times of 24, 48, &72 h, respectively. Ferrocenecarboxaldehyde post-treated group had a GPx activity decline of 20%, 21%, and 16% at periods of 24, 48, & 72 h, respectively. In the group post-treated with malic acid the decline was noted as 16%, 16%, and 15% at periods of 24, 48,& 72 h, respectively (Figure 2C).

##### 4.1.1.4 GSH activity

With the treatment of HEX, there was a decrease in GSH activity to 20%, 28%, and 42% at sampling points of 24, 48, &72 h, respectively, in the treatment group compared to the control group. The Group post-treated with folic acid had a GSH activity decline of 12.4%, 15%, and 11.9% at periods of 24, 48, & 72 h, respectively. Group post-treated with ferrocenecarboxaldehyde had a decline in GSH activity of 17.2%, 28.7%, and 18% at sampling points of 24, 48, & 72 h, respectively. The Group post-treated with malic acid witnessed GSH level drop of 15%, 26%, and 16.5% at the same time periods, respectively (Figure 2D).

##### 4.1.1.5 LPO

**Figure 3:**
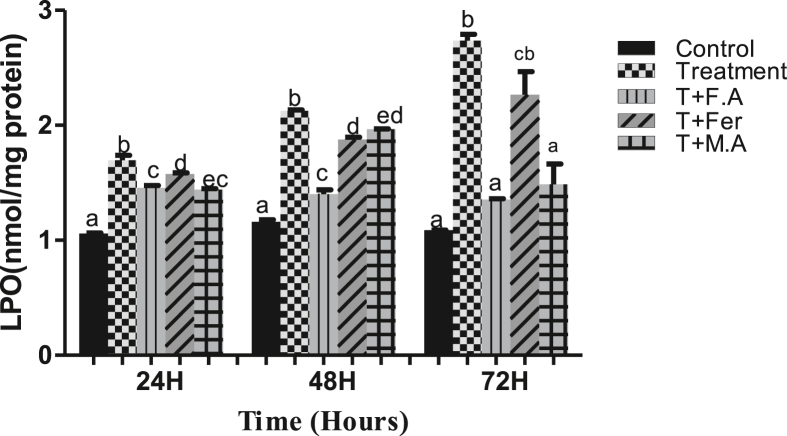
LPO activity of *B. mori* gut after hex treatment and prevention of lipid peroxidation by folic acid, ferrocenecarboxaldehyde and malic acid. The bars with different superscript are significantly different, (P < 0.05).

The treatment group exposed to HEX witnessed increased LPO of 60%, 82%, and 151.5% compared to the control group at sampling points of 24, 48, & 72 h, respectively. The Group co-treated with folic acid showed LPO increasing to 38%, 20%, and 24% at time periods of 24, 48 & 72 h, respectively. Group post-treated with ferrocenecarboxaldehyde showed an increase of 49%, 61%, and 107% in LPO at the same time periods, respectively. The Group co-treated with malic acid showed an increase of 37%, 68%, and 30% in LPO compared to the control group at time periods of 24, 48, & 72 h, respectively (Figure 3).

#### 4.1.2 Fat body

##### 4.1.2.1 CAT activity

**Figure 4:**
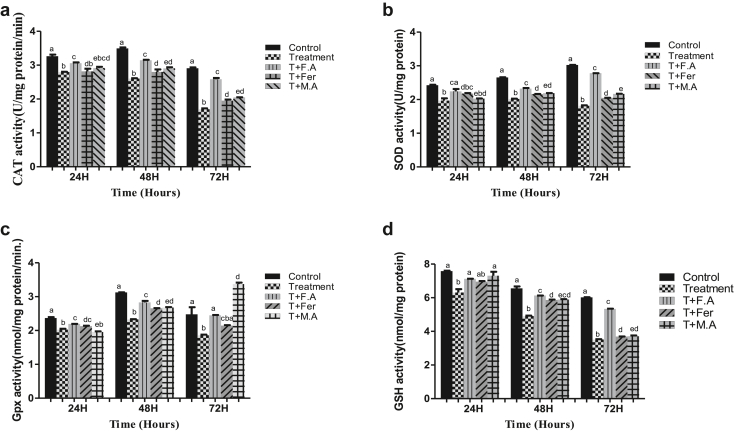
**A**. CAT activity in the fat body of *B. mori* after hex treatment and antioxidant activity of folic acid, ferrocenecarboxaldehyde and malic acid. **B**. bar chart of sod activity following hex treatment and effect of folic acid, ferrocenecarboxaldehyde and malic acid as antioxidant agents, **C**. GPX activity bar graph showing antioxidant activity of folic acid, ferrocenecarboxaldehyde. **D**. GSH activity bar graph showing antioxidant activity of folic acid, ferrocenecarboxaldehyde. The bars with different superscript are significantly different, (P < 0.05).

CAT activity decrease compared to control in the treatment group was 14%, 25%, and 41% at sampling points of 24, 48, & 72 h, respectively. In the group post-treated with folic acid, the decline was 6.4%, 10%, and 11% at time periods of 24, 48, & 72 h, respectively. Group post-treated with ferrocenecarboxaldehyde showed a decline of 14%, 19%, and 32% at time periods of 24, 48, & 72 h, respectively. Group post-treated with malic acid witnessed a CAT activity decline of 10.7%, 16%, and 29% at time periods of 24, 48, & 72 h, respectively (Figure 4A).

##### 4.1.2.2 SOD activity

HEX treatment decreased SOD levels to 18%, 24%, and 40% at sampling points of 24, 48, & 72 h, respectively, compared to the control. Group post-treated with folic acid witnessed a decline of SOD activity to 7.4%, 12%, and 7.9% at the same time periods, respectively. Group post-treated with ferrocenecarboxaldehyde had a 10.3%, 18%, and 32.4% decline in SOD activity at the same time periods, respectively. The Group post-treated with malic acid had a decrease in SOD activity to 16.5%, 17.7%, and 28.4% at sampling points of 24, 48, and 72 h, respectively (Figure 4B).

##### 4.1.2.3 GPx activity

HEX caused a decline in GPx activity of the treatment group, with levels plummeting to 14%, 25%, and 25% at sampling points of 24, 48 & 72 h, respectively, compared to the control group. Group post-treated with folic acid witnessed a decline of 7.2%, 9.2%, and 1.2% at the same time periods of, respectively. Group post-treated with ferrocenecarboxaldehyde had a 10%, 15%, and 13% decline in GPx activity. Group post-treated with malic acid witnessed a decline of 17%, 14%, and 36% at time periods of 24, 48, & 72 h, respectively compared to the control group (Figure 4C).

##### 4.1.2.4 GSH activity

GSH activity with HEX treatment decreased to 16%, 24%, and 41% at sampling points of 24, 48, & 72 h, respectively, compared to the control group. The group receiving post-treatment of folic acid had a GSH decline of 5%, 6.1%, and 11% at the same time periods, respectively. Group post-treated with ferrocenecarboxaldehyde witnessed a decline of 8%, 10.7%, and 40% in GSH activity at the same time periods respectively. The Group post-treated with malic acid saw a drop of 2.6%, 10.7%, and 38% at time periods of 24, 48, & 72 h, respectively compared to the control (Figure 4D).

##### 4.1.2.5 LPO

**Figure 5:**
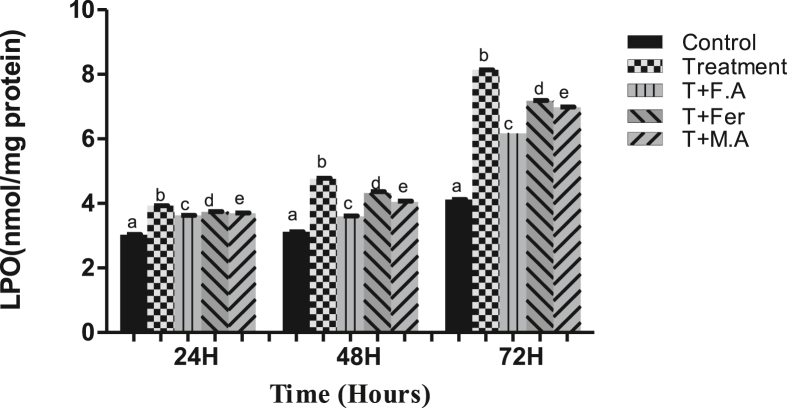
LPO activity of *B. mori* fat body after hex treatment and prevention of lipid peroxidation by folic acid, ferrocenecarboxaldehyde and malic acid. The bars with different superscript are significantly different, (P < 0.05).

LPO increased in the treatment group to 29.3%, 53%, and 97.3% at sampling points of 24, 48, & 72 h respectively compared to the control. In the group post-treated with folic acid, the increase in LPO was 19.4%, 15%, and 49.5% at sampling points of 24, 48 & 72 h, respectively. Group post-treated with ferrocenecarboxaldehyde had an increase in LPO of 23.1%, 38.9%, and 74% at time periods of 24, 48, & 72 h, respectively. The Group post-treated with malic acid had an LPO increase of 21.7%, 33.1%, and 69.1% at time periods of 24, 48, & 72 h, respectively compared to the control group (Figure 5).

#### 4.1.3 Silk gland

##### 4.1.3.1 CAT activity

**Figure 6:**
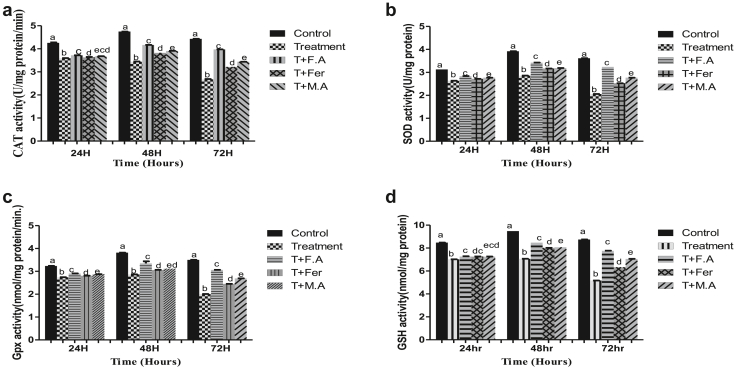
**(A).** CAT activity in silk gland of *B. mori* after hex treatment and antioxidant activity of folic acid, ferrocenecarboxaldehyde and malic acid. **(B**). bar chart of SOD activity following hex treatment and effect of folic acid, ferrocenecarboxaldehyde and malic acid as antioxidant agents, **(C).** GPx activity bar graph showing antioxidant activity of folic acid, ferrocenecarboxaldehyde, **(D).** GSH activity bar graph indicating antioxidant activity of folic acid, malic acid and ferrocenecarboxaldehyde. The bars with different superscript are significantly different, (P < 0.05).

With HEX exposure, CAT activity decreased to 15.2%, 27.6%, and 40% in the treatment group at sampling points of 24, 48, & 72 h, respectively, compared to the control group. The Group post-treated with Folic acid had a CAT activity decline of 12.7%, 12.2%, and 11.3% at the same sampling time periods. The group post-treated with ferrocenecarboxaldehyde showed a decrease of 14%, 12.2%, and 7.2% at the same sampling points. In the group post-treated with malic acid, the decline in CAT activity was 13.6% 17.7%, and 22.7% at sampling points of 24, 48, & 72 h respectively compared to the control group (Figure 6A).

##### 4.1.3.2 SOD activity

At the fixed sampling periods of 24, 48 & 72 h post-treatment, SOD activity in the treatment group dropped to 16%, 28.2%, and 44.4% compared to the control group. The group post-treated with folic acid saw a drop in the SOD activity to 9.6%, 12.8%, and 11.1% at time same sampling time periods. The group receiving ferrocenecarboxaldehyde as post-treatment witnessed a decrease in SOD activity to 12.9%, 20.5%, and 30.5% at already mentioned sampling points. In the group post-treated with malic acid the SOD activity decrease was 12.9%, 20.5%, and 25% at time periods of 24, 48, & 72 h, respectively compared to the control (Figure 6B).

**GPx activity:** HEX induced a reduction of GPx to 15%, 35.7%, and 42.8% at sampling points of 24, 48, & 72 h, respectively compared to the control group. In the group post-treated with folic acid, the reduction was 9.3%, 13.1%, and 14.2%, respectively, at the same time periods. The Group post-treated with ferrocenecarboxaldehyde recorded a GPx activity decline of 12.5%, 21%, and 31.4%, respectively, at the same time periods. In the group post-treated with malic acid, GPx activity got decreased to 12.5%, 18.4%, and 25.7% at time periods of 24, 48, & 72 h, respectively, compared to the control (Figure 6C).

##### 4.1.3.3 GSH activity

HEX caused a decline in GSH activity to 16.6%, 25.5%, and 41.3% in the treatment group at sampling points of 24, 48, & 72 h, respectively, compared to the control group. Group getting a post-treatment of folic acid witnessed a GSH activity decline of 14.2%, 10.6%, and 11.4% at the same time periods, respectively. Group getting post-treated with ferrocenecarboxaldehyde got their GSH activities reduced to 11.9%, 14.8%, and 27.5% at the same time periods, respectively. With malic acid as post-treatment, the group witnessed a GSH activity decline of 14.2%, 14.8%, and 19.5% at the same time periods, respectively (Figure 6D).

##### 4.1.3.4 LPO

**Figure 7:**
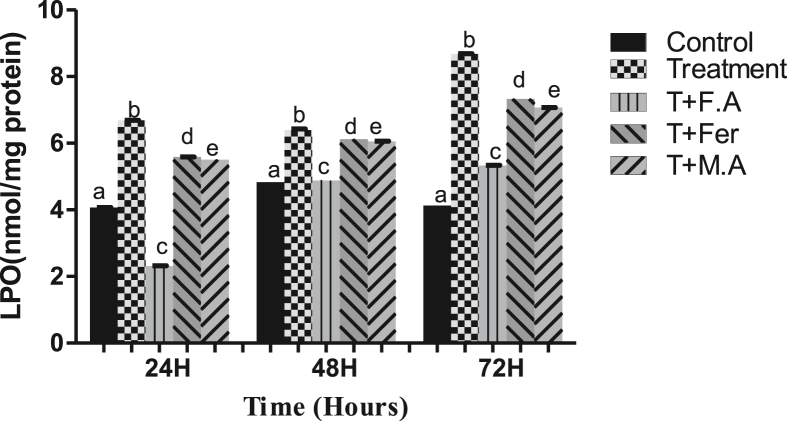
LPO activity of *B. mori* fat body after HEX treatment and prevention of LPO by folic acid, ferrocenecarboxaldehyde and malic acid. The bars with different superscript are significantly different, (P < 0.05).

An increase in LPO to 65%, 31.2%, and 109.7% was seen in the treatment group at sampling points of 24, 48, & 72 h, respectively, compared to the control group. The group receiving folic acid as post-treatment witnessed an increase of LPO to 22.5%, 18.5%, and 29.2% at sampling points of 24, 48, & 72 h, respectively. With ferrocenecarboxaldehyde as post-treatment, the group had an increase in LPO of 37.5%, 27%, and 78% at the time periods of 24, 48 & 72 h, respectively. The group post-treated with malic acid witnessed an increase in LPO of 37.5%, 25%, and 70.7% at time periods of 24, 48, & 72 h, respectively (Figure 7).

## 5 Discussion

**Figure 8:**
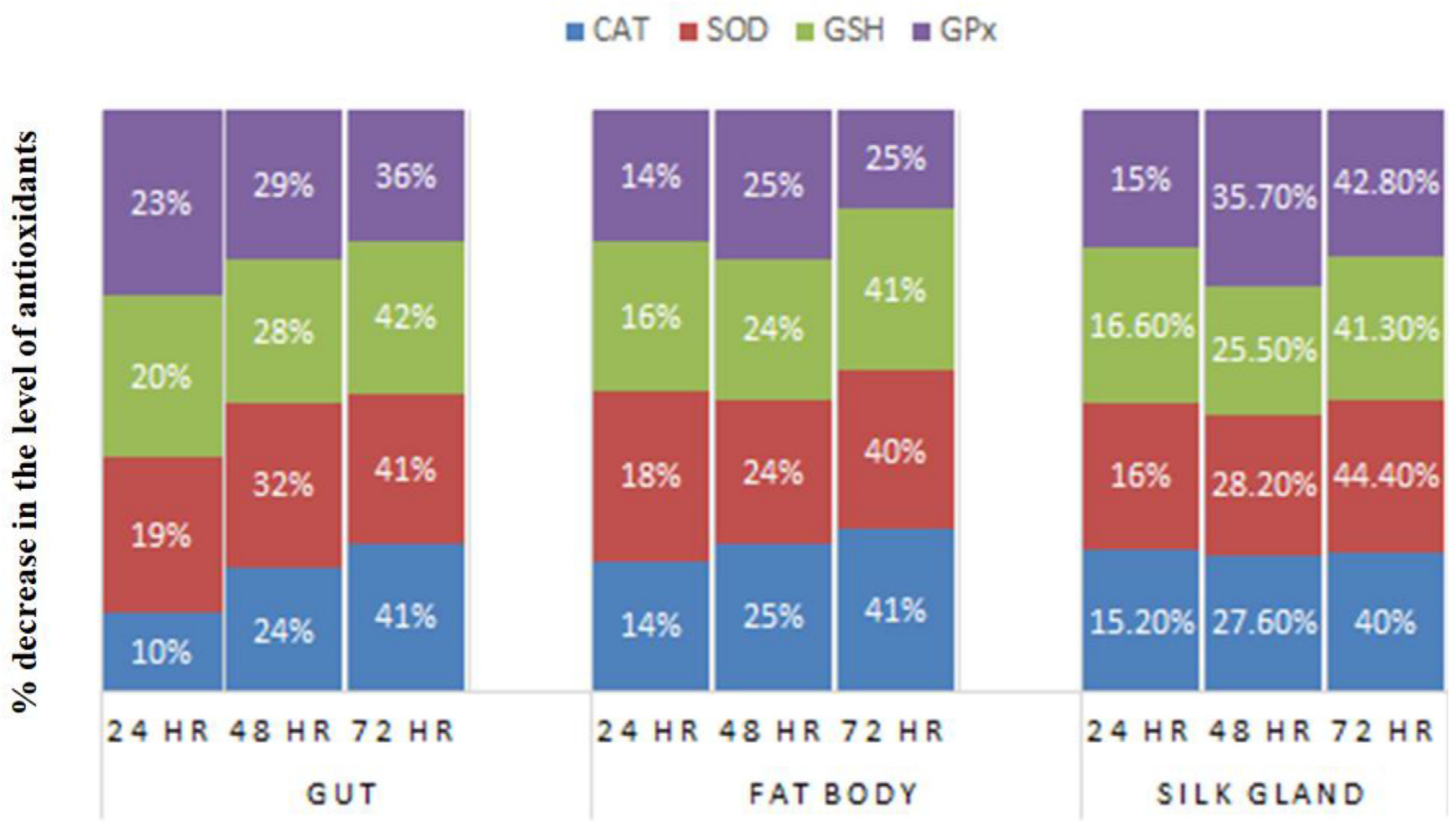
Comparative % decline in antioxidant enzyme (CAT, SOD GSH and GPx) values in gut, fat body and silk gland in silkworm treated with HEX at different time periods.

We started this research to examine the biochemical effects of commercial formulations of HEX, at a sub-lethal concentration (100 mg/l), on the gut, fat body, and silk gland of the invertebrate model *B. mori.* and to evaluate the effectiveness of three different antioxidants, folic acid, ferrocenecarboxaldehyde, and malic acid, on ROS attenuation. The treatment of HEX caused a statistically significant reduction in the levels of antioxidants CAT, SOD, GSH, and GPX in the gut, fat body, and silk gland, which indicated oxidative stress in these tissues. This decrease in antioxidants was a result of the imbalance of the equilibrium between ROS formation and scavenging, leading to increased ROS generation and, therefore, oxidative stress in the tissues. The xenobiotic-induced ROS disbalance has been well studied and is reported as the major cause of oxidative stress generation in the body, which can lead to the beginning of cellular malfunction and death (Mele et al., 2006). Silkworm gut corresponds to the intestine in mammals and is involved in the metabolism process of this organism (Hamamoto et al., 2005; Panthee et al., 2017). A crucial organ for nutrition storage and energy metabolism is the silkworm's fat body (Chen et al., 2018). Due to its similarity to the liver of vertebrates, the fat body of the silkworm is a crucial organ for the metabolism of xenobiotics (Abdelli et al., 2018). The *B. mori* silk gland is a unique organ that produces and secretes proteins, namely fibroin and sericin, two essential ingredients of cocoon silk (Ma et al., 2022). Out of the three tissues under investigation, the gut and fat body showed the greatest reduction in CAT activity and GSH activities. The silk gland showed the greatest reduction in SOD and GPx activity. The fat body was the least impacted of the three tissues comparatively; however still showed a statistically significant drop in the levels of all antioxidants after HEX treatment (Figure 8). The gut tissue displayed the most LPO, followed by the silk gland, while the fat body displayed the least LPO of the three tissues. The different response of different tissues to HEX treatment in the present study is not unusual, and the different response of different organs to the same inducer has been witnessed in other studies too (Q. Li et al., 2019; Liu et al., 2016). According to studies, there are two independent hypotheses that could account for this response specificity: I differential cross-talk across the known pathways, and (ii) new, as of yet unidentified regulators and detoxification mechanisms (Gao et al., 2022).

The results of other investigations on a different model organism, zebrafish, where HEX treatment likewise decreased the antioxidant levels in the body causing ROS stress were consistent with the HEX-induced reduction in antioxidants in the present study (Wang et al., 2015). The most important antioxidants in an organism are CAT and SOD, and a decline in their activity indicates that ROS levels in the cell have increased (Yuan et al., 2018). The decrease in these two enzymes' activity in our study may have been caused by HEX's direct inhibition or by ROS stress altering the enzymes' active sites. The decrease in GSH may be explained by the fact high reducing sulfhydryl groups in GSH regulate complex thiol-exchange. These thiol-exchanges are very important in antioxidant defense (Dickinson and Forman, 2002). Therefore, a decrease in the activity of GSH might be due to the direct oxidation of sulfhydryl groups in GSH by HEX.

The HEX treatment also resulted in LPO in all the tissues under study. The LPO may have been caused by the excessive ROS production giving rise to oxidative stress and, therefore LPO. Free radicals and other oxidants, destroy lipids with carbon-carbon double bonds, especially polyunsaturated fatty acids (Ayala et al., 2014). Other data that lend credence to the hypothesis that oxidative stress is the root cause of LPO point to the possibility that increased oxidative stress is to blame (Lopez-Martinez et al., 2008).

### 5.1 Attenuation of ROS by folic acid, malic acid, and ferrocenecarboxaldehyde

We evaluated the effectiveness of three compounds—folic acid, malic acid, and ferrocenecarboxaldehyde—as ROS scavengers to stop oxidative damage. Numerous studies have demonstrated that using antioxidant chemicals can reduce the ROS stress caused by xenobiotics. Many antioxidant compounds have been able to successfully mitigate ROS stress like eugenol and Coenzyme Q10 in Titanium dioxide (TiO2) nanoparticle toxicity (rats) (Wani et al., 2021; Wani and Shadab, 2021), Luteolin in pesticide Fipronil toxicity (rats) (Seydi et al., 2021). organotellurium and organoselenium compounds in attenuation of Mn-induced toxicity (*C. elegans*) (Avila et al., 2012). Derivatives of some potent antioxidants like caffeic acid have also been found to restore oxidative balance, e.g., Caffeic acid phenethyl ester on adult male Sprague–Dawley rats (Tolba et al., 2017) and Kynurenic as a free radical scavenger in the brain of male Wistar rats (Lugo-Huitrón et al., 2011).

In the current investigation., the malic acid was not able to efficiently stop ROS stress. Malic acid, however did control LPO with statistically significant results. A similar pattern was observed with ferrocenecarboxaldehyde, which also showed poor efficacy in controlling the ROS stress in all tissues under investigation. Studies have shown that ferrocene derivatives have stronger antioxidant capabilities than their ligands (Tabrizi et al., 2020). The efficiency of ferrocenecarboxaldehyde which is a ferrocene derivative in the present study proved to be inconsistent and poor which was contrary to our expectations. The efficiency of this compound in controlling LPO across all tissues was statistically significant, however, here also the efficiency was relatively low compared to other compounds in this study.

Folic acid was the only compound out of the three used in this study that effectively prevented antioxidant levels from falling to a significant level in all of the tissues under study, such as the gut, fat body, and silk gland, as well as prevented and mitigated LPO in all the tissues under study. Folic acid has also shown similar results in other studies dealing with pesticide stress. A meta-analysis study found that folic acid supplementation significantly improves antioxidants (Asbaghi et al., 2021). W. Li et al. (2019) also observed the significance of folic acid in preventing telomeric DNA oxidative damage and telomeric attrition by preventing it from oxidative stress in astrocyte cell culture. Folic acid supplementation also caused a reduction of LPO and an increase in the antioxidant status in mice (Falade et al., 2021).

To scavenge the ROS, antioxidants are considered the solutions and it is technically expected from them to reduce oxidative stress. However, they work in one situation and may fail in another depending on the chemical environment the antioxidant is in (Sharifi-Rad et al., 2020) There have been many instances of failed antioxidant approaches in ROS scavenging e.g. Vit E and C effectiveness in preventing cardiovascular diseases (Sesso et al., 2008; Venditti et al., 2014). Low bioavailability of the antioxidant can be another major factor in determining the effectiveness of the antioxidant. Some antioxidants like polyphenols have been found to have low bioavailability in the blood and tissues (Fernández-García et al., 2012). The low effectiveness of malic acid and ferrocenecarboxaldehyde in the present study may be attributed to these reasons. As a future direction, it would be interesting to see the synergic effect of these antioxidants on the amelioration of oxidative stress and the effect of multiple doses in reducing toxicity. Pesticides are unavoidable today, and toxicities produced from them will occur because of their toxic nature. Therefore, to mitigate this toxicity, antioxidants from natural sources like nutraceuticals should be tried and tested in this field.

## 6 **Conclusion**

HEX caused a decline in the antioxidant enzymes in the silk gland, gut, and fat body of an invertebrate model silkworm *B. mori*. The plummeting in the level of antioxidants leads to oxidative stress which in turn leads to lipid peroxidation causing cellular damage. The oxidative stress was prevented and mitigated by the treatment of a potent antioxidant folic acid efficiently. Ferrocenecarboxaldehyde and malic acid were also used for mitigation, however, they were not efficient enough in preventing ROS stress. Ferrocenecarboxaldehyde was the least effective antioxidant, showing very less antioxidant activity in this study**.**

## Ethical approval

The study needs no ethical approval.

## Consent to participate

The study involves no human trial.

## Declarations

### Author contribution statement

Hashim Ashraf: Conceived and designed the experiments; Performed the experiments; Analysed and interpreted the data; Contributed reagents, materials, analysis tools or data; Wrote the paper.

Ayesha Qamar: Conceived and designed the experiments; Contributed reagents, materials, analysis tools or data; Wrote the paper.

Nikhil Maheshwari: Performed the experiments; Analysed and interpreted the data; Contributed reagents, materials, analysis tools or data.

### Funding statement

This research did not receive any specific grant from funding agencies in the public, commercial, or not-for-profit sectors.

### Data availability statement

Data will be made available on request.

### Declaration of interests statement

The authors declare no conflict of interest.

### Additional information

No additional information is available for this paper.
